# Calcineurin and glial signaling: neuroinflammation and beyond

**DOI:** 10.1186/s12974-014-0158-7

**Published:** 2014-09-10

**Authors:** Jennifer L Furman, Christopher M Norris

**Affiliations:** Pharmacology and Nutritional Sciences and the Sanders-Brown Center on Aging, University of Kentucky College of Medicine, 800 South Limestone St., Lexington, KY 40536 USA; Department of Neurology, School of Medicine, Washington University, 660 South Euclid, St. Louis, MO 63110 USA

**Keywords:** Alzheimer’s disease, Amyloid, Astrocytes, Ca^2+^ regulation, Calcineurin, Gap junction, Glutamate, Neurodegeneration, Neuroinflammation

## Abstract

Similar to peripheral immune/inflammatory cells, neuroglial cells appear to rely on calcineurin (CN) signaling pathways to regulate cytokine production and cellular activation. Several studies suggest that harmful immune/inflammatory responses may be the most impactful consequence of aberrant CN activity in glial cells. However, newly identified roles for CN in glutamate uptake, gap junction regulation, Ca^2+^ dyshomeostasis, and amyloid production suggest that CN’s influence in glia may extend well beyond neuroinflammation. The following review will discuss the various actions of CN in glial cells, with particular emphasis on astrocytes, and consider the implications for neurologic dysfunction arising with aging, injury, and/or neurodegenerative disease.

## Background

Calcineurin (CN) is a Ca^2+^/calmodulin (Ca^2+^/CaM)-dependent protein phosphatase expressed in most mammalian tissues, but found at especially high levels in brain. The CN holoenzyme is a heterodimeric protein containing a 60 kDa catalytic subunit (CN A) and a 19 kDa regulatory subunit (CN B). Multiple isoforms of both subunits have been identified and are expressed differentially throughout brain and most other tissues (for review see [1-3]). CN A contains the catalytic core as well as binding sites for the CN B subunit and Ca^2+^/CaM. There is also a critical autoinhibitory domain located near the C terminus of CN A that suppresses catalytic activity when Ca^2+^ levels are low. The CN B subunit contains four Ca^2+^-binding EF-hand motifs and is generally physically bound to CN A at resting Ca^2+^ levels. Allosteric interactions between CN B, Ca^2+^/CaM, and the autoinhibitory domain allow CN to respond to rapid Ca^2+^ fluctuations with relatively high fidelity [4,5]. Within the cell, CN can be found throughout the cytosol and the nucleus, and is also commonly associated with membrane receptors, ion channels, and pumps via physical interactions with a variety of anchoring proteins [1,3].

Immunohistochemical and *in situ* hybridization studies on healthy adult rat brain performed in the mid-1980s to early 1990s reported high levels of CN in neurons of the striatum, hippocampus, amygdala, and neocortex [6-9], with little to no expression observed in glial cells [6,7]. Many functional studies performed around this time found that CN plays an integral role in coupling glutamate receptor activation to the regulation of cytoskeletal proteins and dendritic spine morphology [10,11]. Since these initial studies, CN has been shown to interact with numerous neuronal substrates and to modulate diverse cellular functions including receptor and ion channel trafficking, ion channel function, apoptosis, and gene regulation, to name a few [2]. In the last 10 years, there have been a steadily increasing number of studies identifying neuronal CN as a primary suspect in synapse loss, dendritic atrophy, synaptic dysfunction, and neuronal vulnerability [12,13]. Nevertheless, despite the apparently selective association of CN with neurons and neuronal signaling cascades, a handful of reports in the mid- to late 1990s found that CN can also appear in primary glial cells and glial cells of intact brain tissue, notably following inflammatory insult [14-17]. The clear connection between glial cells and neuro-immune/inflammatory signaling, in addition to the well-defined role of CN in cytokine production in peripheral immune cells, suggested a strong linkage between glial CN and the neuroinflammation inherent to most acute and chronic neurodegenerative diseases. Recent and ongoing work from our group and others has not only largely confirmed CN as a major modulator of immune/inflammatory processes in glial cells, but has also identified other possible functions for glial CN signaling that may have a major impact on neurologic function. This article reviews the functional implications associated with glial CN expression.

## Review

### CN and glial cells

Though primarily localized to neurons in healthy nervous tissue, CN may also be strongly expressed in glia during aging, injury, and/or disease. Greater numbers of CN-positive astrocytes have been reported in the hippocampus of aged, β-amyloid (Aβ)-bearing transgenic amyloid precursor protein/presenilin 1 (APP/PS1) mice, particularly in the immediate vicinity of extracellular Aβ deposits [18]. A similar relationship between CN-positive astrocytes and Aβ pathology was shown in postmortem brain tissue obtained from human subjects diagnosed with Alzheimer’s disease (AD) [19-21]. Though closely associated with Aβ deposits, the expression of CN in astrocytes does not necessarily depend on the presence of Aβ. Indeed, aged wild-type mice showed a far greater number of CN-positive astrocytes compared to younger wild-type mice, while expression of CN in astrocytes of APP/PS1 mice could be detected as early as three months of age, long before the appearance of extensive plaque pathology in this model [18]. The presence of numerous CN-positive astrocytes in human hippocampus at very early stages of cognitive decline [19] suggests that the upregulation of CN in astrocytes is an antecedent to dementia found at later disease stages. In addition to aging and AD, CN expression in astrocytes has also been reported for animal models of acute injury. In an early study by Hashimoto et al. [16], an increase in astrocytic CN labeling was observed in gerbil hippocampus following bilateral carotid artery occlusion, even though whole tissue levels of CN were reduced (as measured by Western blot). Shifting expression patterns for CN during aging, injury, and disease are notable because of the timing, i.e., CN can appear in astrocytes before widespread pathology is observed, and also because of the selectivity, i.e., increased CN expression only appears to occur in a subset of astrocytes. These findings suggest that changes in CN serve unique and perhaps critical roles in the initiation and progression of neurodegeneration and cognitive decline.

### Phenotype switching in astrocytes and neuroinflammation

Astrocytes are an abundant and diverse subtype of glia. As a critical component of the neurovascular unit, astrocytes ensheath most microvessels via specialized end-foot processes, which help to maintain the integrity of the blood-brain barrier and promote osmotic balance (for review see [22]). Many astrocyte processes are also in close juxtaposition to synapses where they coordinate nutrient exchange to neurons and detoxify the local environment via the uptake of K^+^, glutamate, and other neurotransmitters [23,24]. Redistribution of imported factors and excitotoxins across numerous neighboring astrocytes is accomplished by an extensive gap junction network, which helps minimize local concentration gradients [25]. By ensheathing synapses, astrocytes play an essential role in establishing and maintaining the structural integrity of nerve terminals and dendritic spines, which, in turn, ensures the fidelity of interneuronal communication. In healthy central nervous system (CNS) tissue, astrocytes effectively carry out all of these functions, plus others. However, with CNS injury, astrocytes often look and appear to behave in very different ways. Hypertrophy of astrocyte somata and processes, with a corresponding increase in the expression of the intermediate filament protein, glial fibrillary acidic protein (GFAP), is a pervasive feature of nearly every form of acute CNS injury as well as most chronic neurodegenerative disorders [22,26,27]. These changes have been extensively documented and are commonly referred to as “astrocyte activation” or “astrocyte reactivity” [28].

Along with activated microglia, astrocyte activation is widely accepted as a hallmark of neuroinflammation, though the functional phenotype of activated astrocytes remains somewhat elusive. Protective roles for activated astrocytes, particularly after acute injury, have been demonstrated by many studies (for a review, see [29]). However, if not properly resolved, astrocyte activation can become a chronic condition with apparently detrimental effects on neuronal function and plasticity [28,30]. Activated astrocytes secrete numerous pro-inflammatory cytokines and other factors that can interfere with synaptic fidelity, impair neuronal viability, and/or maintain the activation state of astrocytes and microglia [31-33]. Activated astrocytes also appear to be compromised in their ability to take up excitotoxins (e.g., K^+^ and glutamate) from the extracellular milieu, which could, in turn, lead to further synaptic dysfunction, neuronal damage, and neuroinflammation [19,28,33,34]. The appearance of activated astrocytes at very early stages of cognitive impairment in humans [35-37] suggests that astrocyte activation may help initiate and/or drive other pathophysiological changes leading to dementia.

We have hypothesized that CN is a critical mechanism for triggering phenotype switching (i.e., activated vs. non-activated) and neuroinflammatory signaling inherent to astrocytes during neural damage and dysfunction [13,18,33]. In many peripheral tissues, CN is a pivotal regulator of transcriptional programs involved in cellular remodeling [38,39]. Perhaps the best documented example is the role that CN plays in the adaptive immune response through the activation of NFATs (Nuclear Factor of Activated T cells) and NFκB (Nuclear Factor κB) transcription factors. In the resting state, NFATs and NFκB are both sequestered in the cytosol, albeit by different mechanisms. The nuclear translocation signal of NFATs is masked by hyperphosphorylation. When activated, CN directly binds to and dephosphorylates NFATs, exposing the nuclear translocation signal and promoting the accumulation of NFATs in the nucleus [40]. A similar nuclear localization signal is present in NFκB, but is masked not by hyperphosphorylation, but by the binding of subunits called inhibitory κBs (IκBs) [41]. CN helps promote the activation of IκB kinases [42], which phosphorylate and tag IκB for proteasomal degradation, thereby allowing nuclear translocation of NFκB. Once in the nucleus, NFATs and NFκB interact with distinct DNA binding elements to drive the expression of multiple cytokine species that promote (or in some cases suppress) the clonal expansion of T cells.

In astrocytes, CN appears to represent a fundamental link between morphological changes and immune/inflammatory signaling. Forced expression of activated CN in primary mixed neuron/glia cultures was sufficient to cause an increase in the width of astrocyte somata and processes [18], while inhibition of astrocytic CN/NFAT activity in intact amyloid-bearing mice caused a reduction in the surface area of individual hippocampal astrocytes without affecting overall cell number [43]. Multiple extracellular factors that trigger astrocyte hypertrophy and/or neuroinflammation, including many “pro-inflammatory” cytokines (e.g., interleukin 1β (IL-1β), tumor necrosis factor α (TNFα), and interferon γ), glutamate, ATP, thrombin, S100, and Aβ also robustly activate CN in primary astrocyte cultures [19,21,33,44-48]. Once activated, CN helps drive the expression of numerous immune/inflammatory factors in astrocytes [18,33,44,45], many of which are found at elevated levels during injury, aging, and neurodegenerative disease [49-54]. Moreover, we have found that astrocytic CN/NFAT activity can propagate from one astrocyte population to another in an autostimulatory manner [33].

**Figure 1:**
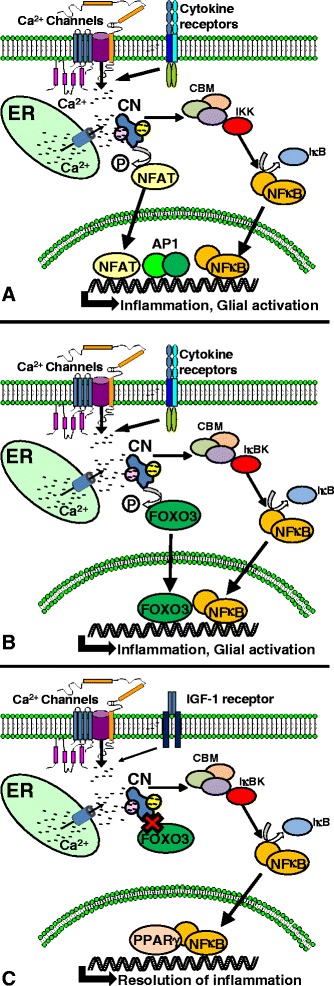
**CN pathways can drive or resolve neuroinflammatory signaling in glial cells. (A)** Factors that activate glial cells, including multiple cytokine species and Aβ peptides, stimulate Ca^2+^ release from endoplasmic reticulum (ER) and/or Ca^2+^ influx across Ca^2+^ channels in the plasma membrane leading to CN activation. CN directly dephosphorylates and activates NFATs. CN also facilitates the recruitment of IκB kinases (IKK) to the CARMA1-Bcl10-MALT1 (CBM) complex, which, in turn, causes the phosphorylation of IκB and the release (i.e., activation) of NFκB. NFATs and NFκB translocate to the nucleus, and with other transcription factors, such as AP1, drive the expression of numerous cytokines involved in the generation and maintenance of neuroinflammation. **(B)** CN can also dephosphorylate and activate FOXO3 transcription factors, which can also synergize with NFκB to drive immune/inflammatory signaling in glial cells. **(C)** Activation of IGF-1 receptors in glial cells also stimulates CN but suppresses its interaction with FOXO3. Simultaneous activation of PPARγ and NFκB by IGF-1 regulates transcriptional programs that reduce or resolve neuroinflammation.

Through its actions on NFAT- and NFκB-dependent transcriptional regulation, CN appears ideally suited to drive the self-perpetuating “cytokine cycles” implicated in chronic neuroinflammation [54,55]. Indeed, similar immune/inflammatory functions of CN have been observed in microglia [56-59], confirming CN’s role as a global mediator of neuroinflammation. Nonetheless, it is probably too simplistic to think of CN strictly as a “pro-inflammatory” mechanism. Extensive work on peripheral immune cells tells us that CN activation can participate in diametrical processes, e.g., cytokine production and clonal expansion under some conditions, and lymphocyte anergy and/or tolerance under different conditions [60]. Such opposing phenotypic characteristics may depend, in part, on the association of CN with a variety of transcription factors. T cell activation, for instance, is largely driven by synergistic actions of NFATs and activator protein 1 (AP1) [40,61] (Figure 1A), while T cell tolerance and/or anergy has been shown to result from interactions between NFATs and forkhead box P3 (FOXP3) [61,62]. Based on these observations in T cells, it is not surprising that CN has been shown to have “anti-“, as well as, “pro-” inflammatory effects in astrocytes. In a series of studies from Torres-Aleman’s group, CN was implicated in both the initiation and resolution of neuroinflammatory signaling in acutely injured mice [45] or mice with progressing amyloid pathology [63]. In the latter study, performed on APP mice expressing a dox-inducible CN fragment in astrocytes, CN was shown to differentially affect neuroinflammation through its interactions with NFκB, peroxisome proliferator-activated receptor γ (PPARγ), and/or forkhead box O3 (FOXO3) transcription factors. These interactions were governed by the presence of specific extracellular factors. TNFα was shown to stimulate interactions between CN, FOXO3, and NFκB leading to increased inflammatory signaling, while insulin-like growth factor (IGF-1) was shown to disrupt the pro-inflammatory interactions between CN, NFκB, and FOXO3 (Figure 1B) in favor of anti-inflammatory interactions between CN, NFκB, and PPARγ (Figure 1C).

Even the presence of different NFAT isoforms can dramatically alter the functional impact of elevated CN activity. A striking example of this is found in muscle tissue where NFAT4 is selectively activated by Ca^2+^ elevations in myoblasts, while NFATs 1 and 2 are selectively activated by the same kind of stimulation in myotubes [64]. In skeletal muscle fibers from adult rats, different NFAT isoforms showed varying sensitivities to different stimulation frequencies and regulated distinct genes associated with slow- and fast-twitch transcriptional programs [65]. The idea that individual NFAT isoforms play various roles in astrocyte function has been proposed by our lab and others [13,19,66-68]. We discovered that early stages of cognitive decline were associated with increased nuclear levels of NFAT1 in astrocytes, while NFAT3 was more strongly associated with astrocytes during late stages of AD [19]. Based on the clear association between the NFAT1 isoform and cytokine expression in lymphocytes, as well as the comparatively weak association of NFAT3 with lymphoid tissues [40], we have suggested that NFAT1 is more strongly linked to the neuroinflammatory phenotype of astrocytes [13]. In contrast, NFAT3 has been more closely linked to cell death and degeneration in multiple cell types (e.g., see [69-71]), suggesting that the demise of astrocytes in severe forms of injury/disease results from the selective activation of NFAT3. The other CN-dependent NFAT isoforms (i.e*.*, NFATs 2 and 4) have also been detected in astrocytes and microglia at the mRNA and protein levels [47,56,59,66-68,72-74]. Several recent studies have reported a striking increase in NFAT4 expression in a subgroup of astrocytes following acute CNS trauma [67,68,73], though the subcellular localization of NFAT4 seemed to be limited to the cytosol, with very little expression found in astrocyte nuclei [67]. Other groups have shown that NFAT4 is indeed active in astrocytes and regulates the expression of transcripts involved in Aβ production [66], as discussed in a later section of this review.

In summary, the available literature suggests that CN plays a major role in shaping the neuroinflammatory phenotype of astrocytes. The impact of CN on neuroinflammation appears to be complex and may depend upon the co-activation of other intracellular signaling pathways and/or the association of CN with multiple transcription factors, including different NFAT isoforms, NFκB, AP1, FOXO3, and others. These differential CN interactions likely do not simply promote a global phenotypic change but instead may selectively affect specific glial functions (Figure 1).

### CN and astrocyte-mediated glutamate regulation

One of the most important functions of astrocytes is the removal of potentially toxic factors from the extracellular milieu, including K^+^, glutamate, GABA, and many others. Glutamate is cleared by astrocytes using a variety of excitatory amino acid transporters (EAATs), which energetically couple glutamate uptake to the import of Na^+^ ions. The EAAT2 isoform (or Glt 1) is expressed predominantly in astrocytes and plays a lead role in glutamate clearance from many brain regions [75]. Loss of EAAT expression and/or function has been linked to a variety of neurologic diseases including AD, amyotrophic lateral sclerosis, Parkinson’s disease, and epilepsy, among others (for reviews, see [24,75,76]). Elevated amyloid levels in human AD brain tissue and in AD animal models is associated with a loss of EAAT expression and/or function [19,77-83]. This loss can occur very early in the progression of cognitive deficits [19], suggesting that changes in glutamate regulation may be key to the initiation/propagation of neuronal degeneration and death. Common phenotypic characteristics of multiple disease models, including increased susceptibility to excitotoxicity, altered synaptic plasticity, and impaired cognition can be recapitulated in otherwise healthy animals by genetically or pharmacologically knocking down EAAT function [84-86]. These observations and many others make EAATs an intensely studied molecular target for preventing and/or limiting neurologic deficits associated with injury and disease.

**Figure 2:**
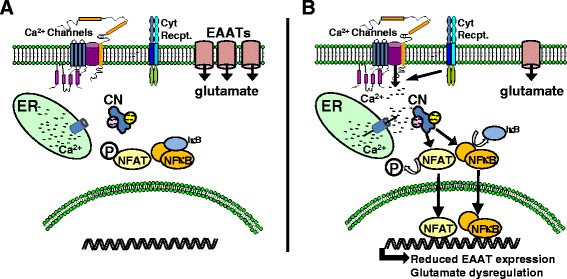
**CN-mediated impairment of glutamate uptake in astrocytes. (A)** In healthy nervous tissue, astrocytes remove excess glutamate from the extracellular milieu using EAATs. **(B)** Pro-inflammatory cytokines and Aβ cause hyperactivation of CN in astrocytes leading to the activation of NFAT and NFκB transcription factors. Together, and possibly individually, NFATs and NFκB suppress the transcription of EAATs. Loss of EAAT protein is associated with reduced glutamate uptake, higher extracellular glutamate levels, and increased excitotoxicity.

Changes in EAATs appear to be strongly linked to the activated astrocyte phenotype. Immunohistochemical analyses of postmortem human brain sections revealed an inverse correlation between EAAT2 and GFAP expression levels [87]. Moreover, multiple extracellular factors that trigger profound astrocyte activation (including pro-inflammatory cytokines and Aβ) can cause a reduction in EAAT expression and/or function in cell culture and animal models [19,33,77,83,88-90]. Numerous mechanisms have been proposed for regulation of EAATs at the transcriptional, translational, and post-translational levels. Many of these mechanisms show strong sensitivity to immune/inflammatory signaling factors implicated in astrocyte activation [91]. In regard to transcriptional regulation, binding sites for NFκB and NFAT have been verified in the human EAAT2 promoter [92]. Interestingly, NFκB appears to play critical roles in both the up- and down-regulation of EAAT2 expression levels. Exposure to a variety of protective/reparative factors, including epidermal growth factor and TGF-β, causes an NFκB-mediated increase in EAAT2 expression, while pro-inflammatory factors like TNFα reduce EAAT2 levels in an NFκB-dependent manner [92]. In our work on primary astrocytes [19,33], reductions in EAAT2 levels following treatment with IL-1β or oligomeric Aβ, were strongly inhibited by pretreatment with VIVIT, an NFAT inhibitory peptide. In addition to preserving EAAT levels, VIVIT lowered extracellular glutamate, dampened neuronal hyperactivity, and reduced the appearance of excitotoxic neuronal death. Given the relatively close proximity between the NFκB and NFAT binding sites within the EAAT2 promoter [92], we suggest that the downregulation of EAAT2 during astrocyte activation and/or neuroinflammation is largely attributable to the synergistic actions of CN, NFκB, and NFAT (Figure 2). Additional work will be required to determine the viability of this combinatorial mechanism for EAAT2 regulation, and to identify possible conditions in which NFATs positively regulate EAAT2 expression to promote glutamate uptake.

### CN, astrocytes, and gap junctions

Astrocytes are extensively coupled to one another and, to a lesser degree, to other cell types via gap junctions (GJ). The GJ channel consists of a hexamer of specialized proteins called connexins (Cx), which are localized to the cell membrane and directly apposed to a similar Cx hexamer localized to the plasma membrane of another cell [25]. The channel formed from apposing connexin hexamers (i.e., Cx hemichannels) can accommodate the passage of molecules up to ~1.2 kDa and provides a direct cytoplasm-to-cytoplasm pathway for adjacent astrocytes. GJs permit the exchange of numerous small molecules and signaling factors between cells including ions, nucleotides, and amino acids. Passage of critical second messengers across GJs, such as Ca^2+^ and inositol trisphosphate (IP3), also allow groups of astrocytes to respond in synchrony to highly localized extracellular signaling factors, which, in turn, can influence the plasticity of local synaptic ensembles and/or modulate the tone of the local vascular network [93]. In addition to forming GJs, some Cx hemichannels are unapposed (i.e., not coupled to Cx hemichannels on other cells) and provide a direct path between the astrocyte cytoplasm and the extracellular milieu. Unapposed Cx hemichannels are generally found in a closed channel state, but may become permeable during brain injury and disease [25].

The diffusion of K^+^ and glutamate across GJ-coupled astrocyte networks during high levels of neuronal activity is one of the fundamental mechanisms for preserving high fidelity interneuronal communication and for preventing excitotoxicity [94]. Transgenic mice lacking Cx43 and/or Cx30 (i.e., the major connexin subtypes found in mature astrocytes) show heightened vulnerability to seizure activity and neuronal death, and may exhibit a variety of neurologic alterations (e.g., altered motor function and impaired cognition) depending on the brain region targeted [95-97]. On the other hand, increased GJ permeability may expose otherwise healthy astrocytes to toxic signals carried from sites of injury or pathology. In this way, GJs have been suggested as a mechanism for spreading pathophysiology [98].

Similar to EAATs, astrocytic GJs are highly sensitive to extracellular factors linked to neuroinflammation and exhibit altered expression and/or function in a variety of different neurologic disorders and diseases [94,99]. Multiple pro-inflammatory cytokines have been shown to either reduce Cx43 expression levels or reduce GJ coupling [100]. The C terminus tail region of Cx43, in particular, contains numerous phosphorylation sites that are regulated by inflammation-sensitive protein kinases [101]. Several research groups have also demonstrated stimulus-evoked dephosphorylation of Cx43 in primary astrocytes, suggesting the dynamic involvement of protein phosphatases [102,103]. Specific dephosphorylation of ser368 in the Cx43 cytoplasmic tail can occur within minutes following a hypoxic insult or after treatment with endogenous factors linked to vascular damage and astrogliosis and is highly sensitive to CN activation [102,103]. Dephosphorylation of ser368 in primary astrocytes following hypoxia or treatment with extracellular factors, such as endothelin-1 and phingosine-1-phosphate, was mostly prevented by pretreatment with commercial CN inhibitors (i.e., cyclosporine A and FK506), supporting a regulatory role for CN in Cx43 dephosphorylation. Additionally, preliminary studies in our laboratory indicate that CN mediates the dephosphorylation of Cx43 ser368 in primary astrocytes exposed to IL-1β or the exogenous Ca^2+^ mobilizing agents ionomycin and phorbol ester [104]. Parallel investigations of Cx43 in postmortem human hippocampal tissue performed by our group further showed that Cx43 dephosphorylation (at ser368) is increased at early stages of cognitive decline and positively correlated with the levels of a proteolytically active form of CN, suggesting that the CN/Cx43 interaction may have relevance to the progression of neurodegeneration and/or dementia.

**Figure 3:**
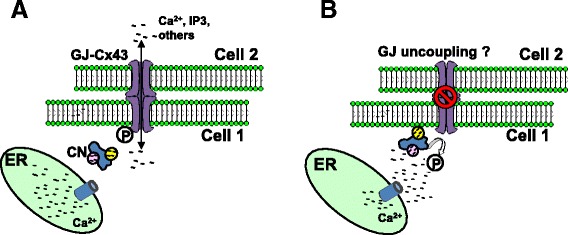
**CN dephosphorylates Cx43 and modulates GJ coupling in astrocytes. (A)** Cx hexamers on the astrocyte membrane form GJ channels with apposing Cx hexamers in adjacent cells and allow the intercellular passage of small molecules like Ca^2+^ and IP3. **(B)** Activation of astrocytes with inflammatory mediators stimulates CN, which dephosphorylates the cytoplasmic tail of Cx43. Dephosphorylation of Cx43 is associated with reduced GJ coupling, though it is unclear whether dephosphorylation is a cause or consequence of decoupling.

Despite these observations, the functional impact of the CN/Cx43 interaction is not presently clear. Using a dye-coupling approach, CN inhibitors were initially shown to prevent the decoupling of GJs in astrocytes following a hypoxic insult [102]. Later studies similarly demonstrated a relationship between GJ inhibition and dephosphorylation of Cx43 ser368, but found that dephosphorylation does not necessarily cause GJ decoupling [103]. Instead, this report suggested that Cx43 dephosphorylation by CN occurs after GJ decoupling and leads to the redistribution of Cx43 to tight junctions. Clearly, additional studies are needed to clarify the role of CN in the regulation of gap junctions and determine whether this interaction significantly impacts astrocyte function in particular, and neurologic function in general (Figure 3).

### CN and astrocytic Ca^2+^ dysregulation

**Figure 4:**
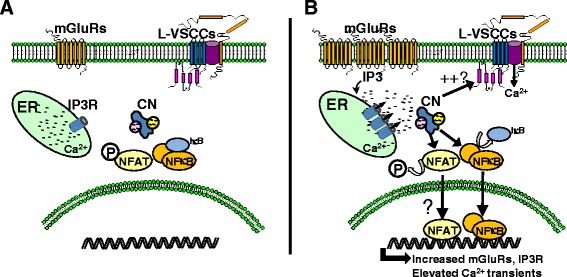
**CN is associated with Ca** ^**2+**^ **dysregulation in astrocytes. (A)** In healthy nervous tissue, elevations in intracellular Ca^2+^ are controlled, in part, by Ca^2+^ release channels (e.g., IP3Rs) located on the endoplasmic reticulum membrane. Levels of several different varieties of plasma membrane Ca^2+^ channels, including L-type Ca^2+^ channels are generally low in non-activated astrocytes. **(B)** Inflammatory mediators, like cytokines and Aβ, cause hyperactivation of CN and its target transcription factors, NFAT and NFκB. These events cause the transcriptional upregulation of IP3 receptors and mGluRs leading to increased Ca^2+^ transients and/or higher resting levels of cytosolic Ca^2+^. CN may also increase the function of L-type Ca^2+^ channels, which, like CN, are found at higher levels in activated astrocytes.

Similar to neurons, activated astrocytes in models of aging, injury, and disease exhibit numerous signs of Ca^2+^ dysregulation including elevated expression of a variety of Ca^2+^ channels and Ca^2+^ regulated proteins, as well as higher intracellular Ca^2+^ levels and/or more frequent Ca^2+^ oscillations [105-107]. While it seems clear how these changes could help set the stage for hyperactive CN signaling, it is also important to note that CN may play an active role in promoting or disrupting Ca^2+^ homeostasis. At the transcriptional level, CN helps drive the expression of many key Ca^2+^ signaling mediators in multiple cell types [108-111]. At the post-translational level, CN directly or indirectly regulates plasma membrane Ca^2+^ channels, intracellular Ca^2+^ release channels, and Ca^2+^-dependent enzymes [2]. In astrocytes, inhibition of CN was recently shown to reduce Ca^2+^ transients evoked by Aβ [21] and to suppress the upregulation of critical proteins involved in Ca^2+^-induced Ca^2+^ release, including IP3 receptor 1 and metabotropic glutamate receptor 5 [112]. Other potential targets for CN may be predicted from findings gleaned from neurons and other cell types. For instance, in neurons and cardiomyocytes, CN has been shown to enhance the function of L-type Ca^2+^ channels, which may, in turn, disrupt cellular activity and viability [113-116]. Though found at very low levels in astrocytes of healthy animals, L-type Ca^2+^ channels appear to be present at high levels in activated astrocytes following acute injury [117]. This differential pattern of expression in astrocytes is strikingly similar to that of CN. Moreover, we have shown that 50% or more of CN/NFAT activity in primary astrocytes treated with IL-1β is eliminated by co-treatment with the L-type Ca^2+^ channel blocker nifedipine [33], while others have shown that CN/NFAT signaling is stimulated in astrocytes treated with the L-type Ca^2+^ channel activator Bayk8644 [44]. These observations suggest that CN/L-type Ca^2+^ channel interactions may play a critical role in promoting Ca^2+^ dysregulation in activated astrocytes (Figure 4).

### CN and astrocyte-derived Aβ production

In AD, CN expression and activity levels are directly correlated to increasing levels of the Aβ peptide [19]. Aβ deposits in both human and mouse tissue are often surrounded by astrocytes that label very intensely for CN [18-20]. The sensitivity of CN activity to rising Aβ levels has been well-documented by several different research groups using a variety of experimental models. Application of the pathogenic, oligomeric form of Aβ rapidly and robustly activates CN signaling pathways in both neurons [71,118-123] and astrocytes [19,21,66,112] and leads to numerous deleterious neurologic changes including enhanced synaptic depression, impaired synaptic potentiation, glutamate dysregulation, dendritic atrophy, cell death, and cognitive deficits.

**Figure 5:**
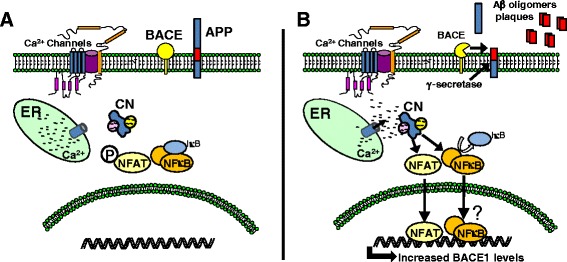
**CN regulates Aβ metabolism in astrocytes. (A)** In resting astrocytes, BACE expression and activity levels are relatively low. **(B)** Activation of astrocytes with inflammatory mediators stimulates CN. Nuclear translocation of NFATs and possibly NFκB leads to the transcriptional upregulation of the protease BACE1 (the rate limiting enzyme for production of neurotoxic Aβ peptides). Elevated levels of BACE1 are associated with the increased production of Aβ peptides, which further aggregate and form Aβ plaques.

Interestingly, APP/PS1 mice treated with FK506 exhibit reductions in Aβ plaque load relative to vehicle-treated APP mice [124], suggesting that CN not only responds to elevated Aβ, but also actively contributes to the accumulation of Aβ pathology. Though the mechanisms through which CN modulates Aβ levels are unsettled, several studies indicate that the CN/NFAT signaling pathway positively regulates the expression of beta-secretases [66,125], the rate-limiting enzymes for formation of Aβ peptides (Figure 5). Jin et al. [66] showed that the NFAT4 isoform binds to the beta-site APP cleaving enzyme 1 (BACE1) promoter in primary astrocytes, which in turn, helps drive Aβ production in response to rising intracellular Ca^2+^ levels. Similarly, our group showed that inhibition of astrocytic NFAT activity reduces soluble Aβ levels and plaque load in the hippocampus of APP/PS1 mice [43] in parallel with a reduction in BACE1 protein levels. We did not observe any changes in the levels for several key Aβ clearing enzymes, including neprilysin and insulin degrading enzyme. Together, these findings are consistent with the notion that astrocytic NFAT/BACE1 interactions play an important role in amyloid regulation. Although BACE1 expression is very low in astrocytes of intact animals and humans, the sheer abundance of this cell type, relative to other cell types, could provide enough BACE activity to contribute significantly to the production of amyloid peptides and subsequent formation of parenchymal Aβ deposits [126]. Nonetheless, effects of CN/NFAT on other key regulators of Aβ formation and clearance, including gamma secretases, apolipoprotein E, and lipoprotein receptor-related protein 1, among others, are not clear and will require further investigation.

In addition to directly influencing Aβ production in astrocytes, it is perhaps just as likely that astrocytic CN indirectly influences Aβ production/metabolism in neurons. Indeed, numerous “pro-inflammatory” factors released by glial cells, many of which are sensitive to CN activity, have been shown to stimulate neuronal APP and/or Aβ production, e.g., see [127-131]. Similarly, the loss of any number of protective glial properties during chronic activation/neuroinflammation would be expected to disrupt Ca^2+^ homeostasis in neurons and/or erode neuronal viability leading to greater Aβ levels [132-136]. Whether direct or indirect, these observations suggest that inhibition of astrocytic CN activity may be an effective strategy for slowing the progression of Aβ pathology.

### Impact of astrocytic CN on neurologic function

Numerous studies have shown that commercial CN inhibitors dampen glial activation, impart neuroprotection, and/or improve neurologic function in animal models of aging, injury, and disease [3,12]. Surely, at least some of these beneficial effects are attributable to direct inhibition of deleterious neuronal CN signaling pathways, which have been shown to play important roles in neuronal degeneration and altered synaptic function (e.g., see [105-107]). However, what about the impact of glial CN signaling? This is a difficult question to address in intact animals using basic pharmacologic reagents, given their lack of cellular specificity. To overcome this difficulty, our group recently employed an adeno-associated virus-based approach to selectively express the NFAT inhibitor, VIVIT, in hippocampal astrocytes of AD model mice [43]. Suppression of astrocytic NFAT signaling in pre-symptomatic mice was sufficient for reducing glial activation and Aβ plaque pathology during advanced age. Arguably more important, this knockdown also proved beneficial to neurologic function and plasticity, i.e., VIVIT-treated mice showed improved synaptic strength, increased levels of long-term potentiation, and better avoidance learning relative to AD mice treated with vehicle or control adeno-associated virus vectors. These observations support a detrimental role for astrocytic CN/NFAT signaling in neurologic function and are consistent with cell culture studies that report lower levels of pro-inflammatory cytokines, reduced Aβ production/lower BACE1 activity, reduced Ca^2+^ transients, lower extracellular glutamate levels, reduced neuronal excitability, and less excitotoxic neuronal death following inhibition of astrocytic CN/NFATs as discussed in preceding sections.

In addition to directly suppressing NFAT activity, overexpression of VIVIT may help to divert CN activity to other substrates that are more protective in nature. For instance, Fernandez et al. [63] reported that overexpression of an activated form of CN in astrocytes of intact APP mice led to the increased association of CN with PPARγ and NFκB, which, in turn, reduced glial activation and amyloid pathology and improved cognition. Together with our findings, it is tempting to speculate that astrocytic CN is generally protective in astrocytes, unless it interacts extensively with NFAT transcription factors, in which case the astrocyte phenotype becomes harmful, marked by increased expression of pro-inflammatory cytokines, impaired glutamate uptake, and other deleterious properties.

## Conclusions

We are just beginning to understand the contribution of CN to glial function. While it seems clear that CN is intimately involved in neuroinflammation, much work is still needed to fully assess the specific interactions between CN-dependent and -independent transcription factors, and how these interactions regulate specific inflammatory phenotypes. While this review has focused primarily on the role of CN in astrocytes, other work showing similar roles of CN in microglia [56-58,74] underscores the necessity to characterize the signaling properties of CN in other glial subtypes. Furthermore, the identification of other cellular targets of CN, including glutamate transporters, gap junctions, and BACE, suggests that CN’s impact on glial function may extend well beyond immune/inflammatory signaling. These observations highlight some of the complexities facing future research into the role of glial CN signaling, but also hint at the potential of discovering new molecular targets for treating neural injury and neurodegenerative disease.

## Abbreviations

Aβ: β-amyloid
AD: Alzheimer’s disease
AP1: Activator protein 1
APP: Amyloid precursor protein
BACE1: Beta-site APP cleaving enzyme 1
CaM: Calmodulin
CN: Calcineurin
CNS: Central nervous system
Cx: Connexins
EAAT: Excitatory amino acid transporter
FOXO3: Forkhead box O3
FOXP3: Forkhead box P3
GJ: Gap junction
GFAP: Glial fibrillary acidic protein
IGF-1: Insulin-like growth factor
IκB: Inhibitory κ B
IL-1β: Interleukin 1β
IP3: Inositol triphosphate
NFATs: Nuclear factor of activated T cells
NFκB: Nuclear factor κ B
PPARγ: Peroxisome proliferator-activated receptor
PS1: Presenilin 1
TNFα: Tumor necrosis factor α
